# Vasoactive intestinal peptide and cystic fibrosis transmembrane conductance regulator contribute to the transepithelial calcium transport across intestinal epithelium-like Caco-2 monolayer

**DOI:** 10.1371/journal.pone.0277096

**Published:** 2022-11-18

**Authors:** Mayuree Rodrat, Kannikar Wongdee, Jarinthorn Teerapornpuntakit, Jirawan Thongbunchoo, Duangrudee Tanramluk, Ratchaneevan Aeimlapa, Nithipak Thammayon, Natchayaporn Thonapan, Pathnaree Wattano, Narattaphol Charoenphandhu

**Affiliations:** 1 Faculty of Science, Center of Calcium and Bone Research (COCAB), Mahidol University, Bangkok, Thailand; 2 Faculty of Science, Department of Physiology, Mahidol University, Bangkok, Thailand; 3 Institute of Molecular Biosciences, Mahidol University, Nakhon Pathom, Thailand; 4 Center of Research and Development for Biomedical Instrumentation, Institute of Molecular Biosciences, Mahidol University, Nakhon Pathom, Thailand; 5 Faculty of Allied Health Sciences, Burapha University, Chonburi, Thailand; 6 Department of Physiology, Faculty of Medical Science, Naresuan University, Phitsanulok, Thailand; 7 Integrative Computational BioScience (ICBS) Center, Mahidol University, Nakhon Pathom, Thailand; 8 Faculty of Science, Graduate Program in Molecular Medicine, Mahidol University, Bangkok, Thailand; 9 The Academy of Science, The Royal Society of Thailand, Bangkok, Thailand; RWTH Aachen University Medical Faculty: Rheinisch-Westfalische Technische Hochschule Aachen Medizinische Fakultat, GERMANY

## Abstract

Vasoactive intestinal peptide (VIP) as a neurocrine factor released by enteric neurons has been postulated to participate in the regulation of transcellular active calcium transport across intestinal epithelium, but the preceding evidence is scant and inconclusive. Herein, transepithelial calcium flux and epithelial electrical parameters were determined by Ussing chamber technique with radioactive tracer in the intestinal epithelium-like Caco-2 monolayer grown on Snapwell. After 3-day culture, Caco-2 cells expressed mRNA of calcium transporters, i.e., TRPV6, calbindin-D_9k_, PMCA_1b_ and NCX1, and exhibited transepithelial resistance of ~200 Ω cm^2^, a characteristic of leaky epithelium similar to the small intestine. VIP receptor agonist was able to enhance transcellular calcium flux, whereas VIP receptor antagonist totally abolished calcium fluxes induced by 1,25-dihydroxyvitamin D_3_ [1,25(OH)_2_D_3_]. Since the intestinal cystic fibrosis transmembrane conductance regulator (CFTR) could be activated by VIP and calciotropic hormones, particularly parathyroid hormone, we sought to determine whether CFTR also contributed to the 1,25(OH)_2_D_3_-induced calcium transport. A selective CFTR inhibitor (20–200 μM CFTR_inh_-172) appeared to diminish calcium fluxes as well as transepithelial potential difference and short-circuit current, both of which indicated a decrease in electrogenic ion transport. On the other hand, 50 μM genistein—a molecule that could rapidly activate CFTR—was found to increase calcium transport. Our *in silico* molecular docking analysis confirmed direct binding of CFTR_inh_-172 and genistein to CFTR channels. In conclusion, VIP and CFTR apparently contributed to the intestinal calcium transport, especially in the presence of 1,25(OH)_2_D_3_, thereby supporting the existence of the neurocrine control of intestinal calcium absorption.

## Introduction

It has long been postulated that neurocrine mediators abundantly produced in the enteric nervous system (ENS)—particularly vasoactive intestinal peptide (VIP)—are able to regulate epithelial calcium transport in the mammalian intestine [1]. VIP appears to exert its effect via receptor called VIP/pituitary adenylate cyclase-activating polypeptide receptors (VPAC). Blais and coworkers [2] reported that intestinal epithelium-like Caco-2 monolayer exposed on the basolateral side to 10 nM VIP exhibited an increase in cellular calcium accumulation, but the calcium transport rate was paradoxically decreased. Several factors might contribute to this discrepancy, for example, low free-ionized calcium concentration, osmolality and pH [3]. Therefore, up until now, there has been no direct evidence to demonstrate the positive effect of VIP on intestinal calcium transport.

Under normal conditions, the transepithelial active calcium absorption across enterocytes occurs in both small and large intestine, but predominantly in the duodenum and cecum, and is positively regulated by 1,25-dihydroxyvitamin D_3_ [1,25(OH)_2_D_3_]. 1,25(OH)_2_D_3_ activates every step of the transcellular calcium absorption, i.e., apical uptake via transient receptor potential vanilloid subfamily member 6 (TRPV6), calbindin-D_9k_-mediated cytoplasmic translocation and active extrusion by plasma membrane Ca^2+^-ATPase 1b (PMCA_1b_) [4,5]. Our previous investigation also showed that the transcellular calcium transport was dependent on the extracellular pH [3]. Specifically, slightly alkaline pH in the vicinity of epithelial cells, especially from HCO_3_^–^, was favorable to calcium absorption, whereas acidic pH similar to that observed during acidosis diminished calcium transport. Thus, the 1,25(OH)_2_D_3_-induced calcium transport probably required the presence of ion channels responsible for bulk HCO_3_^–^ efflux, e.g., cystic fibrosis transmembrane conductance regulator (CFTR) [6].

Recently, we have provided evidence by using single-channel patch clamp and electrophysiological technique to show that opening of CFTR and its activities pertaining to the apical HCO_3_^–^ secretion across the intestinal epithelial cells were enhanced by calciotropic parathyroid hormone (PTH) [7]. PTH is also responsible for enhancing the renal conversion of 25-hydroxyvitamin D_3_ to 1,25(OH)_2_D_3_ [8]. Additionally, VIP is another potent activator of CFTR-mediated intestinal HCO_3_^–^ secretion in a protein kinase A-dependent manner [9].

The present study, therefore, aimed to investigate the contributions of VIP and CFTR during 1,25(OH)_2_D_3_-induced transcellular active calcium transport in Caco-2 monolayer by using ^45^Ca radioactive tracer. After being grown on a permeable membrane (e.g., Snapwell) for at least 3 days, Caco-2 monolayer is able to develop TER as high as 200–250 Ω cm^2^, which is comparable to the previous report of Buzza et al. [10]. The epithelial monolayer with TER of <250 Ω cm^2^ is suitable to represent the leaky epithelia such as proximal small intestine [11], where calcium absorption predominantly occurs *in vivo*. Moreover, the 3-day Caco-2 cells exhibit microvillous formation and sucrase-isomaltase activity [12,13]—both are proxy indicators of absorptive cell differentiation—and strongly express calcium-transporting proteins, e.g., TRPV6 [14,15].

## Materials and methods

### Cell culture

Caco-2 cells [American Type Culture Collection (ATCC) no. HTB-37] were grown in Dulbecco’s modified Eagle’s medium (Sigma, St. Louis, MO) supplemented with 15% fetal bovine serum (GIBCO, GrandIsland, NY), 1% L-glutamine (GIBCO), 1% nonessential amino acid (Sigma), and 100 U/ml penicillin-streptomycin. Cells were propagated in 25-cm^2^ T flasks under a humidified atmosphere containing 5% CO_2_ at 37°C. Culture medium was changed every 2 days and cells were passaged weekly or when 80% of the cells were confluent. Confluent monolayers were prepared by seeding cells on polyester Snapwell with 12-mm diameter and 0.4-μm pore size (catalog no. 3801; Corning) at 4.2 × 10^5^ cells/well. Culture medium was changed after 48-h seeding. Monolayers were incubated at 37°C for 3 days before they were used for calcium flux study.

### Chemicals

VPAC agonist (catalog no. 1911) and VPAC inhibitor or VPAC inh. (catalog no. 1905) were purchased from Tocris Bioscience (Bristol, UK). CFTR_inh_-172 (catalog no. C2992), genistein (catalog no. G6649), and forskolin (catalog no. F6886) were purchased from Sigma. Stock solution of 2.4 mM 1,25(OH)_2_D_3_ (catalog no. 71820; Cayman Chemical, MI, USA) was prepared in 9:1 propylene glycol-ethanol before being diluted to 10 μM by 9:1 propylene glycol-ethanol.

### Experimental design

#### Experiment 1

Caco-2 cells were grown on Snapwells for 3 days. On the experimental day, each Snapwell containing Caco-2 monolayer was mounted into Ussing Chamber and equilibrated with normal bathing solution with no calcium gradient between the two sides of the epithelium. After 10-min equilibration, VPAC agonist was directly added into basolateral solution and incubated for 30 min before calcium flux measurement was performed.

#### Experiment 2

Caco-2 monolayers were pretreated with 10 nM 1,25(OH)_2_D_3_ for 72 h. Thereafter, each Snapwell was transferred into Ussing chamber without transepithelial calcium gradient. Prior to calcium flux measurement, 10 nM 1,25(OH)_2_D_3_-treated monolayer was exposed on the basolateral side for 30 min to 1 or 10 μM VPAC inh.

#### Experiment 3

Caco-2 monolayers were pretreated with 10 nM 1,25(OH)_2_D_3_ for 72 h, and then transferred into Ussing chamber for calcium flux measurement. To determine the effect of CFTR activity on calcium transport, the apical side of the monolayers was exposed to CFTR inhibitor (2–200 μM CFTR_inh_-172).

#### Experiment 4

Caco-2 monolayers were acutely incubated with 50 μM genistein (a direct CFTR activator) on the apical side for 30 min prior to calcium flux measurement. In some experiments, monolayers were also incubated with a combination of 50 μM genistein and 10 μM forskolin (an indirect CFTR activator through cAMP production) on the apical side for 30 min prior to determination of calcium flux after the maximum CFTR activation.

### Real-time polymerase chain reaction (PCR) and immunofluorescence

To confirm that Caco-2 cells were able to express mRNA of calcium transporters, i.e., TRPV6, calbindin-D_9k_, PMCA_1b_ and Na^+^/Ca^2+^-exchanger 1 (NCX1), total RNA extraction and cDNA synthesis were conducted in 3-day Caco-2 cells. Quantitative real-time PCR was performed by QuantStudio 3 Real-Time PCR system (Applied Biosystems, MA, USA), as previously described [16], with specific primers listed in S1 Table. GAPDH was used as a housekeeping gene for normalization, and changes in mRNA levels were calculated from the threshold cycles [16].

Regarding immunofluorescence, Caco-2 cells were plated at 420,000 cells/well on glass coverslips in 24-well plates (Corning, NY, USA). Cells were fixed by 4% paraformaldehyde and nonspecific binding was blocked by 30 min with blocking solution [5% normal goat serum, 4% bovine serum albumin in phosphate-buffered saline (PBS)]. Cells were then incubated overnight at 4°C with 1:100 rabbit anti-TRPV6 primary antibody (catalog no. SC-28763; Santa Cruz Biotechnology, CA, USA) and 1:500 anti-PMCA1 primary antibody (catalog no. ab190355; Abcam, MA, USA). For negative control, cells were incubated only with blocking solution. After being washed, TRPV6 was detected by incubating with 1:500 goat anti-rabbit IgG conjugated with Dylight 594 (catalog no. DI-1594-1.5; Vector Laboratories, CA, USA), while PMCA1 was detected by incubating with 1:500 goat anti-rabbit IgG conjugated with Dylight 488 (catalog no. DI-1488-1.5; Vector Laboratories) for 1 h in dark condition and then mounted by using anti-fade mounting medium with DAPI (catalog no. S36964; Thermo Fisher Scientific, MA, USA). Images were captured using a fluorescent microscope (model Eclipse Ni-U; Nikon, Tokyo, Japan) with cellSens imaging software (Olympus, Tokyo, Japan) and a confocal laser-scanning microscope (model Zeiss LSM800; Carl Zeiss AG, Germany) processed with Zeiss ZEN Blue software.

### Measurement of calcium flux by radioactive tracer

The physiological bathing solution for the Ussing chamber experiments contained (in mM) 118 NaCl, 4.7 KCl, 1.1 MgCl_2_, 1.25 CaCl_2_, 23 NaHCO_3_, 12 D-glucose, and 2 mannitol (Sigma). The solution, continuously gassed with humidified 5% CO_2_ in 95% O_2_, was tightly maintained at 37°C and pH 7.4 and had osmolality of 290–293 mmol/kg H_2_O as measured by a freezing point-based osmometer (model 3320; Advanced Instruments, Norwood, MA, USA). Water used in the present study had electrical resistance >18.3 MΩ∙cm and free-ionized calcium concentration <2.5 nM.

Caco-2 monolayers were mounted in Ussing chamber, in which both apical and basolateral compartments were filled with normal bathing solution. Cells were equilibrated with normal solution for 10 min. Thereafter, it was filled with fresh bathing solution in the basolateral side, whereas the apical side was filled with ^45^CaCl_2_-containing solution (0.45 μCi/mL; final specific activity of 360 mCi/mol; catalog no. NEZ013; PerkinElmer, Boston, MA, USA). ^45^Ca provided a highly precise method for measuring unidirectional calcium flux (*J*_H→C_; nmol·h^–1^·cm^–2^) from the hot side (H; apical side) to the cold side (C; basolateral side), as calculated by Eqs 1 and 2:

$$J_{H\to C}=R_{H\to C}/(S_{H}\times A)$$ (1)

$$S_{H}=C_{H}/C_{To}$$ (2)

where *R*_H→C_ was the rate of ^45^Ca appearance in the cold side (cpm/h); *S*_H_, specific activity in the t side (cpm/nmol); A, epithelial surface area (cm^2^); C_H_, mean radioactivity in the hot side (cpm); and C_To_, total calcium content in the hot side (nmol). Calcium fluxes in the absence of calcium concentration gradient (i.e., bathing solution in both hemichambers contained equal calcium concentration of 1.25 mM) represented the active calcium transport.

### Quantitative analysis of ^45^Ca radioactivity

^45^Ca radionuclides emit β^−^particles (255 KeV with a half-life of 165 days), which were detected by a liquid scintillation spectrophotometer (Tri-carb 3100TR; Perkin Elmer). To determine ^45^Ca radioactivity, 100-μL sample was mixed with scintillation solution cocktail containing 5 g/L 2,5-diphenyloxazole (primary scintillator), 0.3 g/L 1,4-bis[2-(5-phenyloxazolyl)] (secondary scintillator), 50% vol/vol Triton X-100 and 50% vol/vol toluene.

### Measurement of epithelial electrical properties

The three electrical parameters, namely transepithelial potential difference (PD), short-circuit current (*I*_sc_) and transepithelial resistance (TER), were determined as previously described [17]. Briefly, two sets of salt bridges filled with 2 mol/L KCl and 2% wt/vol agar were used to connect Ag/AgCl half-cells (World Precision Instrument, Sarasota, FL, USA) to Ussing chamber, in which a Caco-2 monolayer was mounted between the two fluid compartments. The PD-sensing electrodes were placed near the monolayer, whereas the *I*_sc_-passing electrodes were placed at the end of each compartment. Each Ag/AgCl half-cell was connected to a preamplifier and current-generating unit of an ECV-4000 system (World Precision Instrument). TER was calculated from Ohm’s equation.

### *In silico* analysis of CFTR_inh_-172 and genistein binding

The CFTR structure obtained from electron cryomicroscopy (cryo-EM) was used as a reference molecule (Protein Data Bank ID: 5UAK.pdb; [18]), and all possible pockets were identified using PrankWeb [19]. The MANORAA platform was used to sketch and obtain protein-ligand interacting motifs based on the three-dimensional coordinates from the Protein Data Bank (PDB) [20]. HyperChem was used to correct bond order and hydrogen atoms of the ligand CFTR_inh_-172 and genistein. Conformational search and energy minimization were performed using Molecular Mechanics based on Steepest Descent algorithm [21]. Biased docking was performed by using Biovia Discovery Studio and Autodock Vina 1.2.0 [22,23].

### Statistical analysis

Unless otherwise specified, results were expressed as means ± standard errors of means (SEM). Two sets of independent data were compared by unpaired Student’s *t*-test. One-way analysis of variance (ANOVA) with Tukey’s multiple comparison test was used for multiple sets of data. The significant level for all statistical tests was *P* < 0.05. Data were analyzed by GraphPad Prism 9 (GraphPad Software, San Diego, CA, USA).

## Results

**Fig 1:**
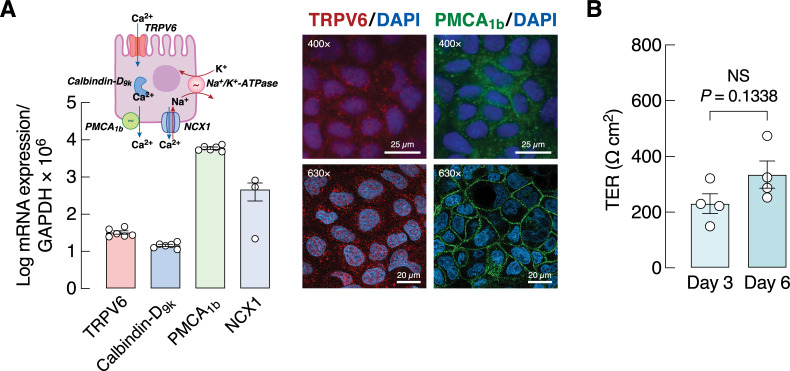
Expression of genes and proteins related to transepithelial calcium transport, and transepithelial resistance in Caco-2 cells. (A) Expression of TRPV6, calbindin-D_9k_, PMCA_1b_ and NCX1 mRNA, as determined by quantitative real-time PCR. Representative fluorescent photomicrographs from conventional fluorescent microscope (400×; Nikon model Eclipse Ni-U) and confocal laser scanning microscope (630×; Carl Zeiss model Zeiss LSM800) confirmed the presence of TRPV6 (red) and PMCA_1b_ (green) protein expression in 3-day Caco-2 monolayer [blue, nuclei stained with 4′,6-diamidino-2-phenylindole (DAPI)]. The drawing diagram shows localization of calcium transporters in a Caco-2 cell. TRPV6, transient receptor potential vanilloid subfamily member 6; PMCA_1b_, plasma membrane Ca^2+^-ATPase 1b; NCX1, Na^+^/Ca^2+^ exchanger 1; NKA, Na^+^/K^+^-ATPase. (B) Transepithelial resistance (TER) of Caco-2 cells after being cultured on Snapwells for 3 or 6 days (n = 4/group; two-tailed, unpaired Student’s *t*-test). NS, not significant.

Prior to investigation of transepithelial calcium fluxes, we used real-time PCR to confirm that 3-day Caco-2 cells were able to express mRNA of TRPV6, calbindin-D_9k_, PMCA_1b_ and NCX1 (Fig 1A). TRPV6 and PMCA_1b_ proteins were visualized by a conventional fluorescent microscope and confocal laser scanning microscope (Fig 1A). After being cultured on a Snapwell for 3 days, Caco-2 cells developed a complete monolayer with TER of ~200 Ω cm^2^ (Fig 1B), and TER was not significantly changed when they were cultured for 6 days (Fig 1B; *P* = 0.1338, two-tailed Student’s *t*-test).

**Fig 2:**
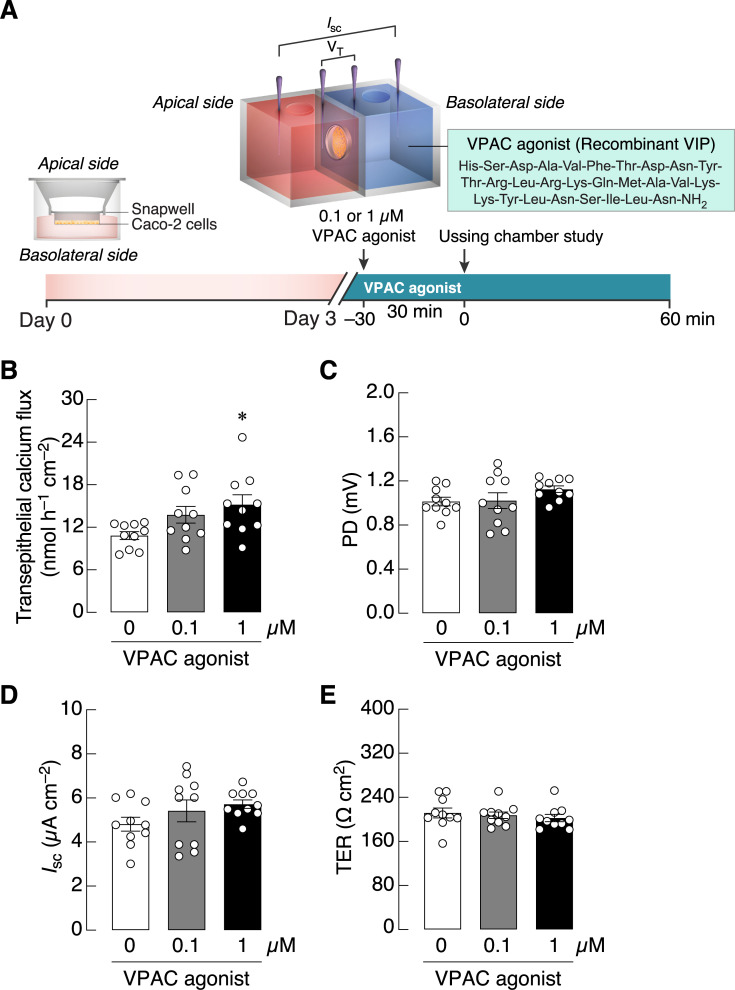
The effects of VIP/pituitary adenylate cyclase-activating polypeptide receptor (VPAC) agonist on intestinal epithelium-like Caco-2 monolayers. (A) Timeline of Experiment 1. (B) Transepithelial calcium flux and (C–E) epithelial electrical parameters (PD, *I*_sc_, and TER) across Caco-2 monolayers that were directly exposed to 0.1 and 1 μM VPAC agonist on the basolateral side for 30 min. *p < 0.05 vs. control group (open bar, one-way ANOVA with Tukey’s multiple comparison test).

**Fig 3:**
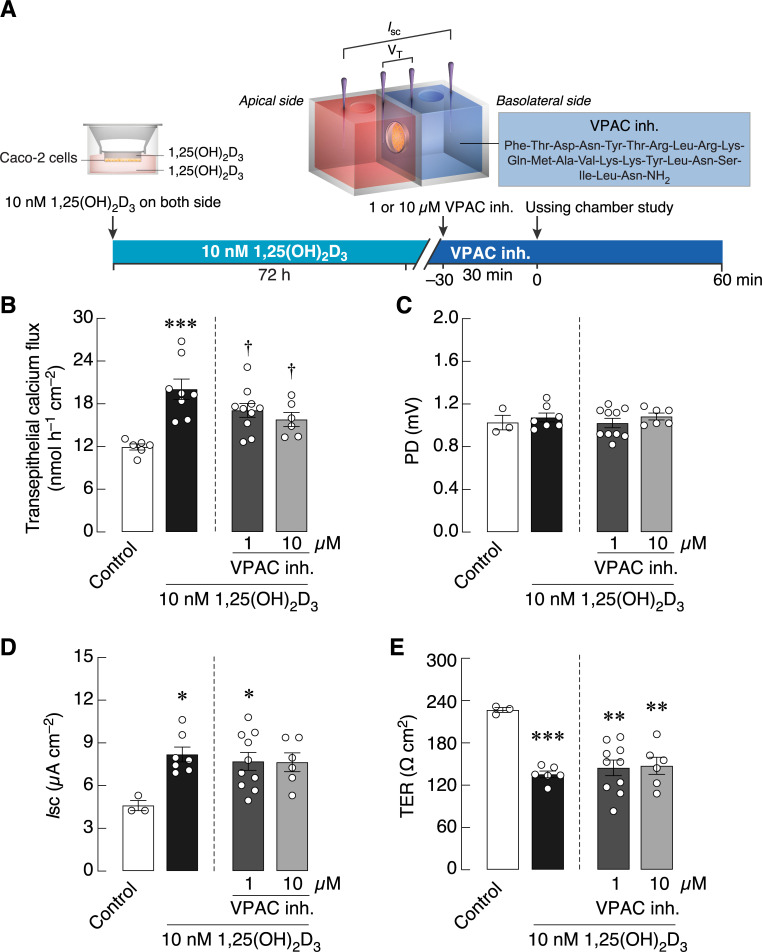
The effects of VIP/pituitary adenylate cyclase-activating polypeptide receptor (VPAC) inhibitor on intestinal epithelium-like Caco-2 monolayers. (A) Timeline of Experiment 2. (B) Transepithelial calcium flux and (C–E) epithelial electrical parameters (PD, *I*_sc_, and TER) across Caco-2 monolayers pretreated with 10 nM 1,25(OH)_2_D_3_ for 72 h and then exposed to 1 and 10 μM VPAC inh. on the basolateral side for 30 min. *p < 0.05; **p < 0.01; ***p < 0.001 vs. control group (open bar, one-way ANOVA with Tukey’s multiple comparison test). ^†^*p* < 0.05 vs. 10 nM 1,25(OH)_2_D_3_-treated group (closed bar, one-way ANOVA with Tukey’s multiple comparison test).

In the first experiment, we determined whether a selective VPAC agonist was able to directly stimulate calcium transport. The results showed that 1 μM VPAC agonist added into the basolateral side of Ussing chamber significantly enhanced transepithelial calcium flux across the Caco-2 monolayer as compared to control (Fig 2; *P* = 0.0199). Since several class-B G-protein-coupled receptors probably had constitutive activities to provide basal support for cellular function [24], we further examined the action of VPAC inh. in 1,25(OH)_2_D_3_-pretreated monolayer (Fig 3A). After treatment with 10 nM 1,25(OH)_2_D_3_, the transepithelial calcium fluxes were markedly increased by ~70%, while 1 and 10 μM VPAC inh. significantly diminished the 1,25(OH)_2_D_3_-induced calcium fluxes (Fig 3), suggesting the basal constitutive activities of VIP receptors. 1,25(OH)_2_D_3_ also altered *I*_sc_ and TER, but VPAC inh. did not modulate the effects of 1,25(OH)_2_D_3_ on both electrical parameters (Fig 3).

**Fig 4:**
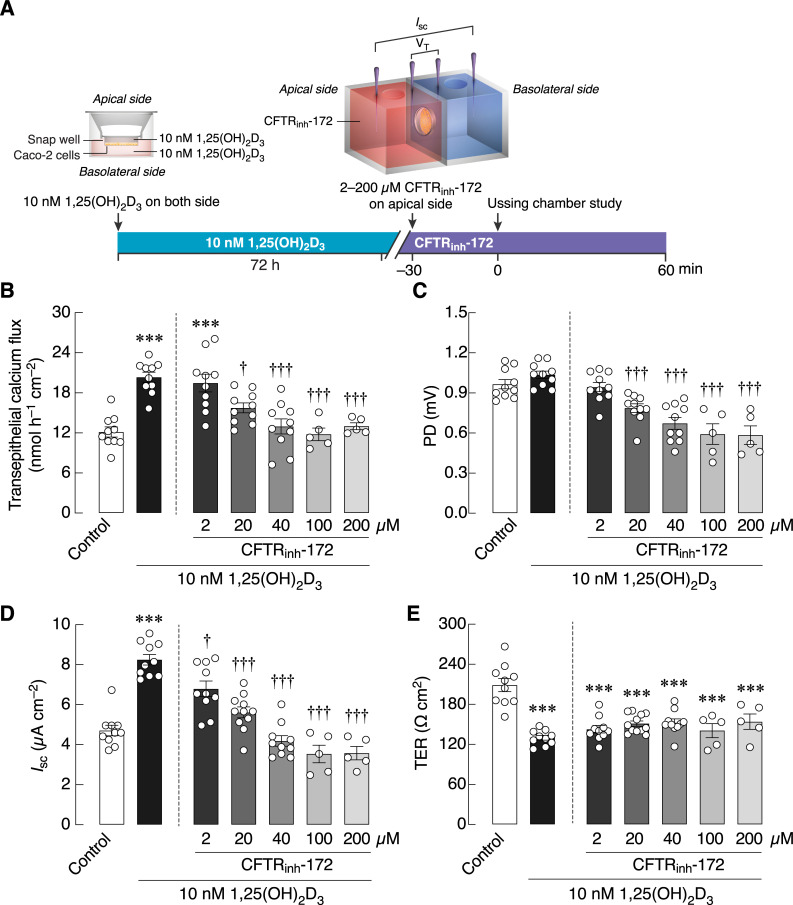
The effects of an CFTR inhibitor on transepithelial calcium transport. (A) Timeline of Experiment 3. (B) Transepithelial calcium flux and (C–E) epithelial electrical parameters (PD, *I*_sc_, and TER) across Caco-2 monolayers pretreated with 10 nM 1,25(OH)_2_D_3_ for 72 h and then exposed to 2–200 μM CFTR inhibitor (CFTR_inh_-172) on the apical side. ***p < 0.001 vs. control group (open bar, one-way ANOVA with Tukey’s multiple comparison test). ^†^*p* < 0.05; ^†††^*p* < 0.001 vs. 10 nM 1,25(OH)_2_D_3_-treated group (closed bar, one-way ANOVA with Tukey’s multiple comparison test).

**Fig 5:**
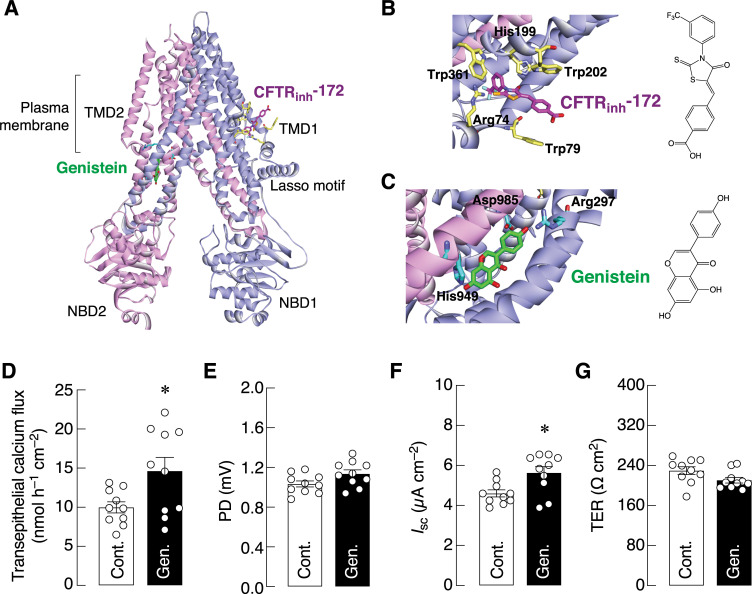
*In silico* molecular docking and the effects of CFTR activator (genistein) on transepithelial calcium transport. (A) The architecture of CFTR and location of the proposed CFTR_inh_-172 (magenta)- and genistein (green)-binding pockets. (B) CFTR_inh_-172 (magenta) surrounded by His199-Arg74-Trp79-Trp202-Trp361 (yellow) resembles the thiazolidinyl ring’s binding interaction from the PDB ID: 4JIR and PDB ID: 4JIH. (C) Genistein (green) was docked to the Arg297-Asp984/5-His949 motif (cyan) in the orientation found in PDB ID: 1X7J. (D) Transepithelial calcium flux and (E–G) epithelial electrical parameters (PD, *I*_sc_, and TER) across Caco-2 monolayers directly exposed to 50 μM genistein (Gen.) on the apical side for 30 min. *p < 0.05 vs. control group (open bar, unpaired Student’s *t*-test).

Although it has been known that exposure of enterocytes to VIP could increase CFTR activity [9], it was unclear if CFTR itself participated in the 1,25(OH)_2_D_3_-induced calcium transport. As shown in Fig 4, a specific CFTR inhibitor, namely CFTR_inh_-172, completely abolished transepithelial calcium fluxes induced by 1,25(OH)_2_D_3_ with a half-maximal inhibitory concentration (IC_50_) of 13.27 μM. Interestingly, CFTR inhibition did not further reduce calcium flux beyond the baseline (control) level. CFTR_inh_-172 also diminished PD and *I*_sc_ (Fig 4), but not TER, indicating that CFTR_inh_-172 had a negative effect on the electrogenic ion transport. Our *in silico* docking analysis further demonstrated that CFTR_inh_-172 possibly inhibited the transporting process through direct binding to CFTR channels, probably at His199-Arg74-Trp79-Trp202-Trp361 (Fig 5A and 5B, S1 File).

Moreover, to confirm the contribution of CFTR in calcium transport, we used genistein that could directly and rapidly potentiate CFTR opening [25]. The present *in silico* docking analysis suggested a high-affinity binding pocket for genistein in the CFTR molecule, presumably at Arg297-Asp984/5-His949 (Fig 5A and 5C, S1 File). In Ussing chamber study, we then acutely exposed Caco-2 monolayer to 50 μM genistein and were able to show that genistein significantly enhanced transepithelial calcium fluxes and *I*_sc_ by 46.54% and 22.32%, respectively, without having effects on PD or TER (Fig 5E–5G). We did not observe any additive effects when a combination of 50 μM genistein and 10 μM forskolin was applied (i.e., mean calcium fluxes of ~14 and 11 nmol h^–1^ cm^–2^ in genistein-treated and genistein+forskolin-treated groups, respectively), suggesting that the maximum calcium transport was attained by 50 μM genistein.

## Discussion

Although the intestinal epithelial cells including Caco-2 cells have been known to express VPAC and CFTR proteins [26,27], contributions of the neurocrine factor VIP and CFTR to the 1,25(OH)_2_D_3_-induced intestinal calcium transport remained enigmatic for decades. In the present study, we demonstrated that VIP was a stimulator of the transepithelial calcium transport and was also essential for the 1,25(OH)_2_D_3_-induced calcium uptake. Although the underlying molecular mechanism remained unclear, it was evident that neural mediator was able to enhance calcium transport, thus supporting the hypothesis of neural control of intestinal calcium absorption.

A number of gastrointestinal functions are tightly regulated by both humoral and neural control. For instance, epithelial secretion of Cl^–^- and HCO_3_^–^-rich fluid via CFTR is enhanced by hormones (e.g., secretin) and neurocrine factors (e.g., VIP, acetylcholine and serotonin) [1,28]. Hence, the intestinal calcium absorption is probably controlled by both modalities, although most investigations have focused on humoral factors—particularly 1,25(OH)_2_D_3_, 17β-estradiol, PTH, insulin and prolactin [1,29]—rather than neural factors. Bone turnover that contributes to calcium homeostasis is also regulated by calciotropic hormones and adrenergic mediators from the autonomic nervous system. It is, therefore, not surprising to find that a factor from the ENS such as VIP was able to modulate intestinal calcium transport.

As mentioned earlier, VIP normally regulates a number of gastrointestinal events, including CFTR activity [9], which is essential for luminal fluidity and pH balance [28]. These known interdependent actions of CFTR in the gastrointestinal system led us to postulate that CFTR also contributed to the intestinal calcium transport. By applying a potent and specific CFTR inhibitor (CFTR_inh_-172), the 1,25(OH)_2_D_3_-induced calcium transport across Caco-2 monolayer was completely abolished (Fig 4). On the other hand, a CFTR activator (genistein) was able to augment calcium transport (Fig 5). Both CFTR_inh_-172 and genistein were found to have novel high-affinity binding sites on CFTR channels, as suggested by *in silico* docking analysis. Kopeikin et al. [30] previously postulated that CFTR_inh_-172 could bind and modulate both open and closed states of CFTR channels, but the exact binding site(s) was not reported therein.

Although it was arguable that genistein might increase calcium transport independent of CFTR—for example through activation of estrogen receptor, which could also increase calcium transport—those mechanisms often required long-term exposure to high concentrations of genistein for several hours or days [31]. Thus, a rapid genistein action as seen in the present study was possibly through direct CFTR activation, similar to that observed in NIH/3T3 fibroblasts [32], rat epididymal epithelium [33], and Fischer rat thyroid gland [34]. Moreover, Wang et al. [35] have provided evidence that genistein could directly bind to CFTR proteins at the nucleotide-binding domain 2 (NBD2), which, in turn, prolonged the opening state of CFTR channels. The present *in silico* analysis also revealed a binding pocket near NBD2, i.e., Arg-Asp-His motif, which has been postulated to be a catalytic triad in enzymatic cleavage of the phosphodiester bond [36]. Furthermore, nearby residues on both sides of His949 agreed well with the scheme of Kubiak et al. [36]—i.e., the plane of the His949 imidazole ring surrounded by Arg289-Glu286-His950, while the proposed area of genistein-binding site aligned longitudinally by Arg297-Asp985-His949. Nevertheless, future experiment is required to visualize genistein-CFTR binding site(s). After binding, genistein probably slowed down dephosphorylation rate of the CFTR regulatory domain, thereby maintaining a steady-state phosphorylation level and prolonging the open state of CFTR channels.

Indeed, the exact mechanism of CFTR action as a crucial player in calcium transport remains elusive. We previously reported that the salient calcium-regulating hormone PTH could directly stimulate CFTR opening, thereby enhancing HCO_3_^–^ transport across Caco-2 monolayer [7,37], while incubating Caco-2 monolayer in HCO_3_^–^-free solution markedly diminished PTH-induced anion transport [38]. Thus, Caco-2 cells used CFTR to transport HCO_3_^–^, which might, in turn, affect calcium transport. In addition, since CFTR_inh_-172 not only abolished calcium transport but also diminished *I*_sc_ induced by 1,25(OH)_2_D_3_ (Fig 4), CFTR was probably essential for 1,25(OH)_2_D_3_ actions rather than for calcium transporters themselves. CFTR opening may indirectly increase calcium transport by altering the extracellular pH in the close vicinity to the apical membrane, where TRPV6 is abundantly expressed. It has been shown that a slight alkaline pH was able to increase transepithelial calcium absorption, whereas metabolic acidosis negatively affected calcium transport [39], presumably due to the fact that H^+^ can compete for the Ca^2+^-binding site on TRPV6, thereby reducing Ca^2+^ influx. Moreover, patients with cystic fibrosis—a genetic disease caused by CFTR gene mutation—have dysregulation of calcium homeostasis including impaired calcium absorption and excretion, as well as low bone mineral density [40,41]. Consistently, several studies also reported that cystic fibrosis patients developed osteopenia and osteoporosis [42,43]. The aforementioned evidence thus helped support the involvement of CFTR in calcium transport.

In conclusions, both VIP and CFTR were found to be essential components for 1,25(OH)_2_D_3_ signaling in enhancing transepithelial calcium transport across Caco-2 monolayer. Although more experiments are required to demonstrate their underlying cellular and molecular mechanisms, the present data have shed some light on the existence of neural control of intestinal calcium transport and have provided foundation for further investigations of how ENS controls epithelial transport of calcium *in vivo*, thus supporting the SDG 3 (Good Health and Well-being) of United Nations Sustainable Development Goals (SDG).

## Supporting information

S1 Table *Homo sapiens* primers used in real-time PCR. The sequences of primers of calcium transport-related genes.
(DOCX)

S1 File Proposed CFTR_inh_-172- and genistein-binding pockets of CFTR channel. A video showing the architecture of a CFTR channel and the sites of proposed CFTR_inh_-172- and genistein-binding pockets (magenta and green, respectively). The movie was created by using PyMOL 1.7.4 (The PyMOL Molecular Graphics System, Schrödinger, LLC.).
(ZIP)
