# Supplementary material for: Vasoactive intestinal peptide and cystic fibrosis transmembrane conductance regulator contribute to the transepithelial calcium transport across intestinal epithelium-like Caco-2 monolayer
Source: PLoS One. 2022 Nov 18;17(11):e0277096. doi: 10.1371/journal.pone.0277096 (PMC9674163; doi:10.1371/journal.pone.0277096)
Supplement: S1 Table — The sequences of primers of calcium transport-related genes. (DOCX) [file pone.0277096.s001.docx]

**S1 Table.** ***Homo sapiens* primers used in real-time PCR.** The sequences of primers of calcium transport related genes.

| **Gene** | **Accession no.** | **Primer (Forward/Reverse)** | **Product size (bp)** | **Annealing temperature (°C)** |
| --- | --- | --- | --- | --- |
| *Calcium transport related genes* | | | | |
| TRPV6 | AF365928 | 5’–TCTGACTGCGTGTTCTCAC–3’  5’–ACATTCCTTGGCGTTCAT–3’ | 144 | 56 |
| Calbindin-D_9k_ | NM_004057 | 5’–TAGCTGTTTCACTATTGGGCA–3’  5’–TTCATCCTTTGACAACTGGTCT–3’ | 127 | 56 |
| PMCA_1b_ | NM_001001323 | 5’–AGAAGGTGGAGATGGTGATGA–3’  5’–CCCAGAAGGTGTCAATGACA–3’ | 179 | 56 |
| NCX1 | NM_001112801 | 5’–TTGCACTCTGTGTTTATGTGGTG–3’  5’–AAGTAAGCAAACCTTCCCAGA–3’ | 168 | 53 |
| *Housekeeping gene* | | | | |
| GAPDH | NM_001289746 | 5’–TTGTTGCCATCAATGACCC–3’  5’–ATTTTGGAGGGATCTCGCT–3’ | 166 | 53 |

TRPV6, transient receptor potential cation channel subfamily V member 6; PMCA_1b_, plasma membrane Ca^2+^-ATPase-1b; Na^+^/Ca^2+^-exchanger 1 (NCX1); GAPDH, glyceraldehyde-3-phosphate dehydrogenase.
